# Primary High-Grade Myxoid Liposarcoma of the Extremities: Prognostic Factors and Metastatic Pattern

**DOI:** 10.3390/cancers14112657

**Published:** 2022-05-27

**Authors:** Gianmarco Tuzzato, Roberta Laranga, Federico Ostetto, Elisa Bubbico, Giulio Vara, Giuseppe Bianchi

**Affiliations:** 1 Unit of 3rd Orthopaedic and Traumatologic Clinic Prevalently Oncologic, IRCCS Istituto Ortopedico Rizzoli, Via Pupilli 1, 40136 Bologna, Italy; gianmarco.tuzzato@ior.it (G.T.); federico.ostetto@ior.it (F.O.); elisa.bubbico@ior.it (E.B.); giuseppe.bianchi@ior.it (G.B.); 2 Department of Radiology, IRCCS Azienda Ospedaliero-Universitaria di Bologna, Via Albertoni 15, 40138 Bologna, Italy; giulio.vara@studio.unibo.it

**Keywords:** liposarcoma myxoid, metastases, high grade, extremities

## Abstract

**Simple Summary:**

Around 12–25% of patients with high-grade myxoid liposarcoma (MLS) develop local relapses and 30–60% develop pulmonary or extrapulmonary metastases over a 10-year follow-up period. The aim of this study was to evaluate factors that may influence overall survival (OS), local recurrence-free survival (LRFS), metastasis-free survival (MFS), post-relapse survival (PRS), and disease-free survival (DFS) in a select series of localized, deep-seated, high-grade myxoid liposarcomas of the extremities. The tumor sites and surgical margins were not found to be significant risk factors for OS, LRFS, MFS, PRS, and DFS in this study, confirming that patients with tumors in the extremities have a more favorable prognosis. Additionally, we concluded that tumor size is a significant risk factor for MFS and DFS.

**Abstract:**

(1) Background: This retrospective study aimed to analyze the history and treatment outcomes of localized, high-grade MLS of the extremities. (2) Methods: We retrospectively reviewed 82 patients with primary high-grade MLS of the extremities. OS, LRFS, MFS, PRS, and DFS were analyzed. (3) Results: Five-year OS and LRS were 96% (95% CI: 86–98) and 94% (95% CI: 85–98), respectively. Statistical analysis indicated no risk factors for OS and LFRS. MFS was 77% (65–85) at 5-year follow-up. Size (*p* = 0.0337) was the only risk factor statistically significant for MFS (HR = 0.248, 95% CI: 0.07–0.84). Median PRS after distant metastasis was 34 months (range: 1–127 months). Five-year PRS was 79% (48–93). Overall, the 5-year DFS was 76% (65–85). (4) Conclusions: Patients with MLS were found to have a good prognosis. In high-grade deep-seated tumors, common risk factors for MLS do not correlate with survival. Tumor size appears to be the only predictor of long-term DSF and MSF.

## 1. Introduction

Liposarcoma—a malignant mesenchymal tumor originating in adipose tissue—is the second most common soft tissue sarcoma, accounting for between 15 and 20% of cases. Of the different variants, myxoid/round cell liposarcoma (MLS) is the second most common subtype (20–30% of cases), following well-differentiated liposarcoma/atypical lipomatous tumor [1]. 

A myxoid matrix with lipomatous differentiation characterizes the histopathology and is clearly distinguishable from the other variants due to the presence of the chimeric gene FUS-DDIT3 [2] or, less commonly, EWSR1-DDIT3 [3,4]. 

It is well known that myxoid liposarcomas and round cell liposarcomas rank in the same class of neoplasms, the round cell form appearing in a higher-grade and poorly differentiated form [4]. Additionally, previous studies have demonstrated that the presence and amount of a round cell variant greater than 5% results in an unfavorable prognosis and distinguishes low-grade from high-grade MLS [5,6,7].

High-grade MLS is highly sensitive to radiotherapy (RT) and partly sensitive to chemotherapy (Cht) [6,8], and treatment is usually a combination of surgery and RT associated with Cht, according to clinical presentation. The literature focusing on the characterization and management of high-grade MLS is currently limited. Most documented reports include either low-grade or high-grade MLS. MLS typically originates in the deeper soft tissue of the extremities, commonly in the leg. It does not show epidemiological differences between the two sexes, and the appearance of MLS peaks between the third and the fifth decade of life [5,9]. Local relapses occur in 12–25% and distant metastases in 30–60% of cases, even years after the initial diagnosis [10,11,12], in multiple interesting and atypical regions. The affected sites are usually the bones, soft tissues, lymph nodes, lungs, and abdomen [9,13].

According to the literature, there are many factors affecting prognosis in MLS, including tumor size and depth, surgical margins, tumor grade, and the use of neoadjuvant or adjuvant therapies [8,14,15]. This study aimed to evaluate factors that may influence overall survival (OS), local recurrence-free survival (LRFS), metastasis-free survival (MFS), post-relapse survival (PRS), and disease-free survival (DFS) in our department.

## 2. Patients and Methods

We retrospectively reviewed the histological and clinical records of 86 patients affected by myxoid liposarcoma treated between 2007 and 2021. From this first cohort, we selected 82 patients with primary, localized, high-grade, deep-seated tumors in their extremities. Follow-up data and patient status at the last follow-up were available and updated to their previous visit through a digital archive. Clinical data included patient characteristics (age, gender), tumor characteristics (site, size, depth, and histology), the diagnostic and therapeutic procedures used (type of surgery, surgical margins, neoadjuvant, and adjuvant therapy), and clinical outcomes (status, local recurrence, and distant metastasis after treatment).

Histological diagnosis was confirmed by open incisional biopsy or ultrasound needle biopsy. Round cell liposarcoma is recognized as a high-grade, cell-specific variant of MLS characterized by poor prognosis. Tumor size was assessed with a pre-operative MRI. Histologic slides were reviewed, and tumors were graded according to the WHO 2020 classifications [16,17].

The surgical approach was chosen according to the possibility of achieving the broadest oncological margins. In the event of adjacency to critical structures (blood vessels, nerves, or bones), planned marginal surgery was accepted. Surgical margins were assessed according to the Musculoskeletal Tumor Society as defined by Ennekin et al. [18]. Wide corresponds to the presence of normal tissue between tumor and margin. Marginal is defined when resection is along the pseudocapsule or reactive zone around the tumor; intralesional for a macroscopic or microscopic tumor at the margin.

RT was delivered with the most appropriate technique available, reaching a total dose of 50 Gy in 1.8–2 Gy fractions in the pre-operative setting. In the post-operative setting, doses up to 66 Gy were given, depending on the presentation, age, and resection margins. The administration of Cht in patients with MLS could be defined as a neoadjuvant for local control of primary MLS, an adjuvant in a post-operative setting, or for the treatment of metastatic MLS. Cht was applied in many cases due to our sarcoma center’s typical treatment regimen for soft tissue sarcomas. Combinations of doxorubicin and ifosfamide or epirubicin and ifosfamide were used as neoadjuvant and adjuvant treatments. For the treatment of metastatic MLS, trabectedin, eventually supplemented with neoadjuvant doxorubicin-based therapy, was the elected therapy. Surgery took place 4–8 weeks after the termination of the last cycle of Cht or the last fraction of RT [19].

At every visit, patients underwent clinical examination during follow-up: every 3 months for the first 2 years, every 4 months during the third year, every 6 months for the fourth to the fifth year, and annually from the sixth to the tenth year. An MRI with contrast enhancement of the primary tumor site and a chest CT scan were performed at every follow-up. An abdominal CT scan with contrast enhancement was performed every 6 months for the first 2 years, every 8 months during the third year, and annually during the rest of the follow-up until the tenth year. After relapse, patients started a new follow-up cycle from the beginning.

### Statistical Methods

Patient characteristics were reported as medians and percentages for continuous and categorical variables, respectively. All variables were analyzed for their impact on OS, LRFS, MFS, PRS, and DFS, with a follow-up of 5 years. OS was calculated from diagnosis to death by any cause. LRFS was calculated from diagnosis to local recurrence onset or death due to the disease. MFS was calculated from diagnosis to metastasis onset to death due to the disease. PRFS was calculated from metastasis onset to death due to the disease. DFS was defined as the length of time, in months, from the date of surgery to the date of last follow-up or the local and distant recurrence.

In a univariate analysis of the OS estimates, LRFS, MFS, PRS, and DFS were calculated according to the Kaplan–Meier method. The log-rank test media performed the calculation and comparison of survival curves. The hazard ratios and confidence intervals (95%) were calculated using the Cox hazard test. Values of *p* ≤ 0.05 were considered statistically significant. The statistical analysis was performed with Stata software version 17.0.

## 3. Results

### 3.1. Clinicopathological Features

Our data included 82 primitive localized high-grade deep-seated myxoid liposarcomas (MLS). Table 1 summarizes characteristics of the patients and the tumors. In our study, we analyzed 53 men and 29 women. The mean age at diagnosis was 50 years (25–76). With regard to localization, the tumors were distributed as follows: 51 (62%) MLS were found in the thigh, 24 (29%) in the leg, 6 (7%) in the buttock, and 1 (1%) in the arm. The pre-operative MRI showed sizes over 10 cm in 50 (61%) patients, between 5 and 10 cm in 29 (35%) patients, and under 5 cm in 3 (4%) patients. All tumors were classified as high-grade (over 5% round cell).

The elective surgery treatment was excision (98% of cases). At the same time, only two patients were treated with amputation due to the tumor’s critical size and location (leg and thigh). Post-operative histology confirmed that 63 (77%) MLS were removed with wide margins, 16 (20%) with marginal margins, and 3 (4%) with intralesional margins. In the cases of amputation (2/86), surgical margins were radical (2%).

Pre-operative RT was performed in 57 MLS (40 cases over 10 cm in size, 14 cases between 5 and 10 cm, and 3 cases with size under 5 cm), while the post-operative RT was performed in 19 patients (7 over 10 cm, 12 between 5 and 10 cm, and 0 under 5 cm) (Table 1). Cht was administered to 60 (73%) MLS patients, while 45 (55%) patients underwent neoadjuvant Cht. Lastly, 13 (16%) patients had post-operative Cht and 2 (2%) patients had both pre- and post-operative treatment (Table 1).

Follow-up data were available for all patients. The median follow-up was 65 months (range: 9–146 months). At the end of the study, 95% of patients were alive, while 5% had died. In the cohort of surviving patients, 68 were alive without disease, and 10 patients were alive with metastases. 

### 3.2. Overall Survival

**Figure 1:**
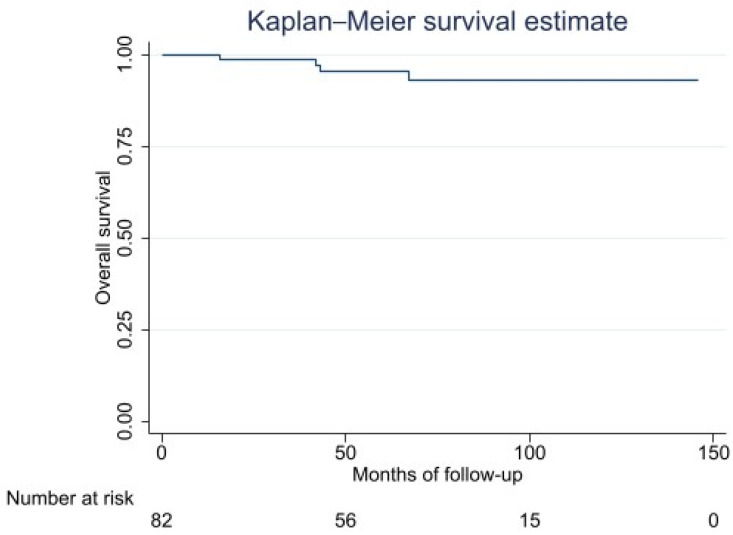
Overall survival Kaplan–Meier curve.

Five-year OS was 96% (95% CI: 86–98) (Figure 1). Statistical analysis indicated that there were no risk factors for OS (Table 2).

### 3.3. Local Recurrence

Local recurrence was reported in four cases (5%), with a median free interval of 3 months (range: 0–32 months). Three out of four cases had a tumors over 10 cm, and one case exhibited a size ranging between 5 and 10 cm. Regarding treatment, two out of the four patients underwent pre-operative RT and all patients were treated with Cht. In all cases, the margin was wide/radical. Two patients were alive without evidence of disease at the last follow-up, one patient was alive with disease, and the last one died because of a distant progression. All local recurrences were re-excised. LRFS was 94% (95% CI: 85–98) at 5 years. Statistical analysis indicated that there were no risk factors for LRFS.

### 3.4. Distant Metastasis: Metastasis-Free Survival, Metastatic Pattern, and Post-Relapse Survival

**Figure 2:**
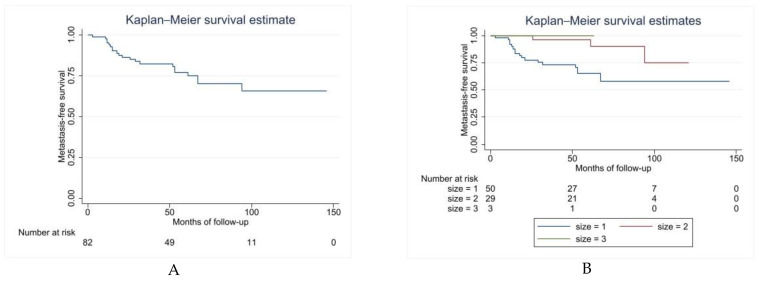
Metastasis-free survival (MFS) Kaplan–Meier curves. (**A**) Overall MFS. (**B**) Kaplan–Meier curve comparing local MFS by size of tumor: size 1 ≥ 10 cm, size 2 = 5–10 cm, size 3 ≤ 5 cm (*p* = 0.0337).

Twenty MLS (24%) developed metastases, with a median free interval of 20 months (range: 3–94 months). MFS was 77% (65–85) at 5 years (Figure 2A). Size (*p* = 0.0337) was the only identified independent risk factor statistically significant for MFS (HR = 0.248, 95% CI: 0.07–0.84) (Figure 2B and Table 3).

The numbers and sites of metastases were one in the abdominal muscular wall (5%), five in bones (25%), two in lymph nodes (10%), three in lungs (15%), and the last nine patients had multiple locations (45%) (most frequently soft tissue, lungs, viscera, lymph nodes). Three out of twenty cases of metastatic MLS had tumors from 5–10 cm; all the others had tumors under 5 cm.

**Figure 3:**
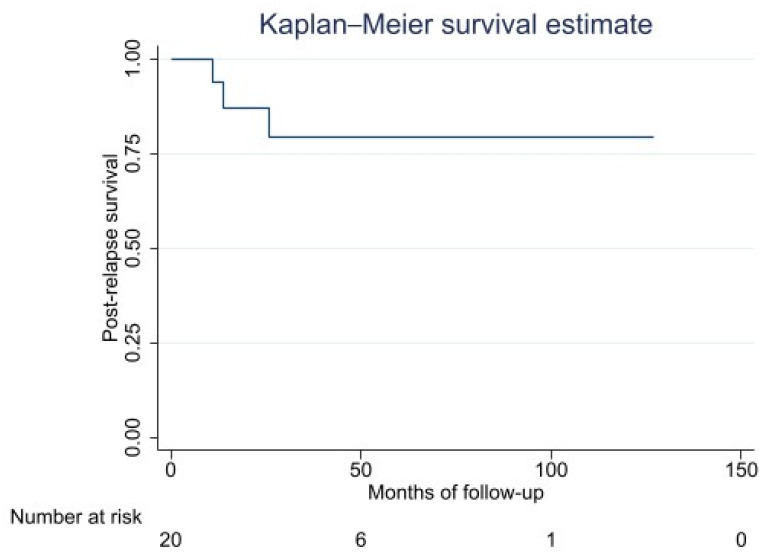
Post-relapse survival estimates.

Among the metastatic patients, 35% showed no evidence of disease following treatment of metastasis, 50% were alive with the disease at the last follow-up and 15% had died. Median PRS was 34 months (range: 1–127 months). Five-year PRS was 79% (48–93 months) (Figure 3).

Metastasis treatment consisted of surgery alone (25%) or surgery combined with RT and Cht (55%). The preferred surgical procedure was excision (90%), though two patients were treated with amputation (one of them in combination with RT and the other with Cht). Four patients (20%) were treated with surgery and RT, and four patients (20%) had surgery plus Cht. The other three patients were treated with RT, surgery, and Cht. Among the 20 metastatic patients, only two were not treated with surgery (10%): one underwent Cht, and another was treated with RT. The last two patients did not undergo treatment (one patient underwent early palliative care, the other refused adjuvant therapies). No risk factors for PRS could be identified.

### 3.5. Disease-Free Survival

**Figure 4:**
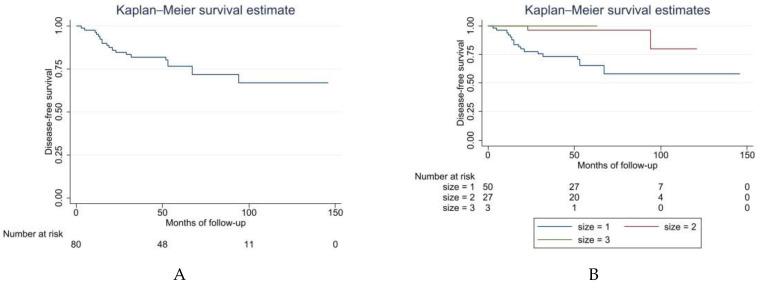
Disease-free survival Kaplan–Meier curve. (**A**) Overall DSF. (**B**) Kaplan–Meier curve comparing DFS by size of tumor: size 1 ≥ 10 cm, size 2 = 5–10 cm, size 3 ≤ 5 cm (*p* = 0.0199).

Altogether, 68 patients (83%) in this study showed no evidence of disease following treatment for local recurrence or metastases. Ten (12%) patients were alive with disease at the last follow-up, and 4 patients died (5%). Sixty-one patients (74%) were DF after primary surgery during the follow-up period. Overall, the 5-year DFS was 76% (65–85) (Figure 4A). We also recognized that size can be a risk factor for DFS (log-rank test *p* = 0.0199; COX proportional Hazard HR = 0.177; 95% CI: 0.04–0.76) (Figure 4B and Table 4).

## 4. Discussion

The study aimed to evaluate factors that may influence OS, LRFS, MFS, PRS, and DFS in high-grade MLS of the extremities. We also analyzed several factors that had a possible impact on the clinical outcomes of patients with MLS in a consecutive single-institution series. Histological grade is an established prognostic factor [7,10,11]. Muratori et al. reported that tumor grade was a significant risk factor affecting the OS of MLS [7]. Similarly, Whu et al. revealed that tumor grade was an independent prognostic factor of OS and cancer-specific survival [20]. In this study, we were not able to confirm these results and audit the effectiveness of histologic grade as a prognostic factor, since our series comprised only deep-seated high-grade tumors. While these limitations created a more homogeneous group for analysis and contributed novelty to the already published reports, they might have reduced the importance of other factors for prognosis.

In this work, the tumoral site and surgical margins were not significant risk factors for OS, LRFS, MFS, PRS, or DFS, confirming that tumors in the extremities have a good prognosis [11]. Different studies have analyzed MLS patients and identified trunk tumor location as being associated with a poorer outcome than an extremity location [10,21,22]. It is also noteworthy that the impact of tumor localization on the overall outcome is also related to the ability to perform resection with adequate surgical margins, as reported in other works on MLS [23,24].

Tumor size is a known prognostic factor for soft tissue sarcomas. According to the published data, the bigger the tumor, the worse the prognosis [25,26,27,28]. In our series, we showed that tumor size is a significant risk factor for MFS (log-rank test *p* = 0.0337, HR = 0.248. 95% CI 0.07–0.84) and DFS (log-rank test *p* = 0.0199, HR = 0.1773 95% CI: 0.04–0.76). These findings are congruent with those of Salduz et al. and Wu et al., who found that tumor size over 15 cm was significantly associated with increased overall mortality and demonstrated that tumor size under 10 cm was an independent prognostic factor of OS and cancer-specific survival [20,29]. In contrast, Nishida et al. and Oh et al. reported that tumor size was not associated with survival [21,22] but that size may influence higher-grade MLS.

Regarding treatment, the role of chemotherapy in patients with soft tissue sarcomas has been investigated extensively [23,30,31], as has the radio sensitivity behavior of MLS [31]. We did not succeed in showing whether chemotherapy or radiotherapy provided a clinical benefit for local recurrence, metastasis-free, post-relapse, or overall survival. However, it is essential to note that the selected employment of chemotherapy in the highest-risk patients confounded this analysis. Further investigation would be needed to help better determine the optimal use of chemotherapy in this group of patients.

Similarly, because most patients (97%) had received radiotherapy, we were not able to show the effect of radiation therapy in our series. There are contradictory opinions on the effects of radiotherapy on survival of extremity MLS patients. Some evidence supports the role of RT in improving survival [32,33]. Others found that RT had no impact on either the OS or DFS of MLS [21]. Additionally, it is well documented that this category of patients could achieve local control by only surgical treatment [11,21]. Further studies are warranted to investigate the effect of RT on the outcome and survival of extremity MLS.

Previous studies have shown that MLS patients display higher survival rates than other soft tissues sarcoma patients [34,35]. We observed a favorable estimated 5-year OS rate of 96%. Estimated OS rate was 96%. However, this is not confirmed in other datasets. We observed a higher rate in comparison to previous results reported by Guadagnolo et al. [30], and Saluz et al. [29] who described a 5-year OS corresponding to the 87% and 78.1% respectively. A possible explanation of this difference may be linked to the length of follow-up and to the number of cases analyzed.

LRFS was 94% (95% CI: 85–98) at 5 years. Despite previous results reported by other authors, our local recurrence rate was lower (5%), possibly due to the inclusion criteria included for our work. Selecting only patients with primary tumors treated for the first time at our institute made it possible to highlight the importance of accurate surgical planning and the involvement of specialized centers. This idea is highly accepted by other authors [10,15]. In addition, as confirmed in previous studies [7,30], this low rate of local relapse could be related to the positive effect of post-operative radiotherapy treatment and chemotherapy treatment, performed in 93% and 60% of the patients, respectively.

Finally, it should not be underestimated that the favorable localization of the tumor in the outer extremities made it possible to intervene with higher precision in resection and surgical margins.

We observed a metastatic rate of 24%; sites of distant metastasis included: bones (25%), lungs (15%), lymph nodes (10%), and more than one involved sites (soft tissue, lungs, viscera, lymph nodes) (45%).The crude rate of distant metastasis seems to be comparable with the published series reporting DM rates of 11–38% [4,5,21]. Moreover, these data confirmed previous results, which showed that MLS has a distinct non-pulmonary metastatic disease pattern [30]. Therefore, patients with high-risk extremity myxoid liposarcoma should undergo imaging studies of the chest, abdomen, spine, and pelvis as part of their staging and follow-up examinations, preferably with full-body MRI, or CT and MRI scans of the spine and pelvic region, for detection of suspected metastatic disease. Of the twenty metastatic patients, 35% had no evidence of disease following treatment of metastasis, 50% were alive with the disease at the last follow-up, and 15% died. Overall, 5-year PRS was 79% (95% CI: 48–93).

DFS is a direct measure of clinical benefit. Regarding our series, 74% of patients were DF after primary surgery at the end of the follow-up period, which indicates a generally favorable prognosis associated with this kind of soft tissue sarcoma. These results are similar (47–73%) to what has been reported in previously published reports [4,36]. Higher rates were reported by Nishida et al., who reviewed 53 patients with extremity and trunk MLS, with a 5-year DFS rate of 90% and a median follow-up time of 60 months [21]. This difference in outcomes between this study and previous reports may mainly reflect differences in tumor locations and the inclusion of the round cell subtype.

The homogeneity of the case series, the interest in choosing only high-grade tumors of the extremities, the absence of missing data, and the treatment of patients at a single institute are all strengths of our study. Moreover, considering that to date our cohort of 82 cases of primary high-grade MLS of the extremities, excluding other subtypes, is the biggest presented from a sole institute, makes an effort in growing the clinical record on this disease. However, some limitations must be acknowledged, the first of which is the relatively small number of patients and the follow-up period. Another limitation is the study’s retrospective design. An adequate control group could not be formed, and confounding variables were challenging to measure, making it hard to make simple statements. 

## 5. Conclusions

Myxoid liposarcoma has a relatively good clinical outcome. Our analysis revealed that by selecting only high-grade, deep-seated tumors, the common risk factors for MLS do not correlate with survival. However, large tumors tend to be more aggressive, and tumor size is the most significant predictor of long-term DFS and MFS. In myxoid liposarcoma, it seems reasonable to expect an adequate local control rate after RT and Cht. Lastly, the distinctive pattern of metastatic relapse seen in MLS necessitates staging and surveillance imaging of the abdomen, pelvis, and the thorax because extrapulmonary soft tissue metastases are common sites for distant metastases.

Our findings have clinical implications for the treatment and surveillance of patients with localized myxoid liposarcoma and could be valuable in the daily clinical practice of oncologists and oncological orthopedic surgeons. 

## Figures and Tables

**Table 1:** Main clinicopathological features and therapeutic approaches.

Factor	Number of Patients	%
Patients	82	100
Gender		
Male	53	65
Female	29	35
Location		
Thigh	51	62
Leg	24	29
Buttock	6	7
Arm	1	1
Tumor size		
>10 cm	50	61
5–10 cm	29	35
<5 cm	3	4
Surgery		
Excision	80	98
Amputation	2	2
Margin		
Wide/radical	63	77
Marginal	16	20
Intralesional	3	4
Radiotherapy		
Pre-operative	57	68
Post-operative	19	23
None	6	9
Chemotherapy		
Pre-operative	45	55
Post-operative	13	16
Pre- and post-operative	2	2
None	22	27

**Table 2:** Kaplan–Maier Overall Survival (OS) estimates from patient characteristics.

Factor	Level	Kaplan–Maier Estimates (95% CI) 5-Year	*p* > chi2
Gender	F	100% (ND)	0.592
	M	93% (80–97)	
Age	>60	100% (ND)	0.787
	<60	94% (83–98)	
Size	>10 cm	93% (79–97)	0.2919
	5–10 cm	100%	
	<5 cm	100%	
Location	Thigh	93% (79–97)	0.8938
	Leg	100%	
	Buttock	100%	
	Other	100%	
Margin	Wide/Radical	96% (85–99)	0.0806
	Marginal	93% (59–99)	
	Intralesional	100%	
Chemotherapy	Yes	94% (81–97)	0.8497
	No	100%	
Radiotherapy	Yes	96% (87–99)	0.1279
	No	83% (27–97)	

95% CI: 95% Confidence Interval; F: Female; M: Male; ND: not determined.

**Table 3:** Kaplan–Maier estimates for MFS from patient characteristics. * Log-rank test. ** HRs unadjusted to each other in multivariable analysis. Results unchanged upon adjustment. (Size adjusted for location, RT and margin: HR = 0.24 (*p* = 0.029; 95% CI: 0.06–0.866)).

Factor	Level	*p* > chi2	HR (95%CI)
Gender	F (ref)	0.1270	
	M		
Age	>60 (ref)	0.8263	
	<60		
Size	>10 cm (ref)	* 0.0337	** 0.248 (0.07–0.84) *p* = 0.026
	5–10 cm		
	<5 cm		
Location	Tight (ref)	0.3708	
	Leg		
	Buttock		
	Other		
Margin	Wide (ref)	0.1099	
	Marginal		
	Intralesional		
	Radical		
Chemotherapy	Yes (ref)	0.0961	
	No		
Radiotherapy	Yes (ref)	0.1161	
	No		

HR: Hazard Ratio; 95% CI: 95% Confidence Interval; F: Female; M: Male; ref: group of reference.

**Table 4:** Kaplan–Maier Disease-free survival (DFS) estimates from patient characteristics. ** HRs unadjusted to each other in multivariable analysis. Results did not change upon an adjustment in multivariate analysis.

Factor	Level	*p* > chi2	HR (95% CI)
Gender	F (ref)	0.1462	
	M		
Age	>60 (ref)	0.7886	
	<60		
Size	>10 cm (ref)	0.0199	** 0.1773 *p* = 0.020 95% CI: 0.4–0.76
	5–10 cm		
	<5 cm		
Location	Tight (ref)	0.3236	
	Leg		
	Buttock		
	Arm		
Margin	Wide/radical (ref)	0.1097	
	Marginal		
	Intralesional		
Chemotherapy	Yes (ref)	0.1081	
	No		
Radiotherapy	Yes (ref)	0.2820	
	No		

HR: Hazard Ratio; 95% CI: 95% Confidence Interval; F: Female; M: Male; ref: group of reference.

## Data Availability

The data presented in this study are available on request from the corresponding author. The data are not publicly available due to privacy or ethical restrictions.
